# Association of dietary patterns, anthropometric measurements, and metabolic parameters with C-reactive protein and neutrophil-to-lymphocyte ratio in middle-aged and older adults with metabolic syndrome in Taiwan: a cross-sectional study

**DOI:** 10.1186/s12937-018-0417-z

**Published:** 2018-11-19

**Authors:** Ahmad Syauqy, Chien-Yeh Hsu, Hsiao-Hsien Rau, Jane C.-J. Chao

**Affiliations:** 1 School of Nutrition and Health Sciences, College of Nutrition, Taipei Medical University, 250 Wu-Hsing Street, Taipei, 110 Taiwan; 2 Department of Nutrition Science, Faculty of Medicine, Diponegoro University, Semarang, Indonesia; 3 Department of Information Management, National Taipei University of Nursing and Health Sciences, Taipei, Taiwan; 4 Master Program in Global Health and Development, College of Public Health, Taipei Medical University, Taipei, Taiwan; 5 Joint Commission of Taiwan, New Taipei City, Taiwan; 6 Nutrition Research Center, Taipei Medical University Hospital, Taipei, Taiwan

**Keywords:** Dietary patterns, Anthropometric measurements, Metabolic parameters, C-reactive protein, Neutrophil-to-lymphocyte ratio, Inflammation, Metabolic syndrome

## Abstract

**Background:**

Metabolic syndrome is commonly associated with inflammation. The underlying factors of inflammation in metabolic syndrome are not fully understood. The objective of the study was to determine the association of dietary patterns, anthropometric measurements, and metabolic parameters with inflammatory markers in middle-aged and older adults with metabolic syndrome in Taiwan.

**Methods:**

A total of 26,016 subjects aged ≥35 y with metabolic syndrome were recruited from Mei Jau institution between 2004 and 2013 for a cross sectional study. Metabolic syndrome was defined by the International Diabetes Federation. Multivariate logistic regression was performed to evaluate the association of dietary patterns, anthropometric measurements, and metabolic parameters with C-reactive protein (CRP) and neutrophil-to-lymphocyte ratio (NLR) in men and women with metabolic syndrome. Crude and adjusted models were analyzed by gender.

**Results:**

The western dietary pattern, obesity, high body fat, high waist or hip circumference, and high waist-to-hip ratio were significantly associated with increased odds ratios of high CRP and NLR in both genders. High systolic or diastolic blood pressure (BP), low high-density lipoprotein-cholesterol (HDL-C), high low-density lipoprotein-cholesterol (LDL-C), high total cholesterol (TC), high serum triglycerides (TG), and high fasting blood glucose (FBG) were significantly correlated with increased odds ratios of high CRP in both genders. Low HDL-C, high LDL-C, high serum TG, and high FBG were significantly associated with increased odds ratios of high NLR in both genders. However, high systolic (OR = 1.124, 95% CI 1.047–1.206, *P* < 0.01) or diastolic BP (OR = 1.176, 95% CI 1.087–1.273, *P* < 0.001) and high TC (OR = 1.138, 95% CI 1.062–1.220, *P* < 0.001) were significantly correlated with increased odds ratios of high NLR only in men.

**Conclusions:**

The western dietary pattern, obese-related anthropometric parameters, and most components of metabolic syndrome are positively associated with CRP levels and NLR in men and women with metabolic syndrome.

## Background

Metabolic syndrome is defined by central obesity, increased systolic and diastolic blood pressure (BP), decreased high-density lipoprotein cholesterol (HDL-C), increased serum triglycerides (TG), and elevated fasting blood glucose (FBG). The International Diabetes Federation (IDF) declared that central obesity was strongly associated with metabolic syndrome and its components [1]. The prevalence of metabolic syndrome has been increased obviously throughout the world [2–4], and it has become a main public health issue in recent years. Moreover, metabolic syndrome is one of the risk factors of cardiovascular disease (CVD). The prevalence of metabolic syndrome and central obesity increased with age, with the highest rates seen among middle-aged and older adults [5]. Additionally, metabolic syndrome was associated with inflammation which may exacerbate the development of CVD [6]. The increased levels of inflammatory markers have also been strongly correlated with both central obesity and metabolic syndrome. However, the underlying factors of inflammation in metabolic syndrome are not fully understood.

Dietary patterns are associated with inflammation. The previous study revealed that high intake of trans fatty acids had a positive correlation with inflammation [7]. Additionally, a diet high in meat and processed food was positively correlated with inflammation [8, 9]. In contrast, higher intake of vegetables was inversely associated with C-reactive protein (CRP) concentrations [10, 11]. Anthropometric parameters were also correlated with inflammation. Obesity defined by body mass index (BMI) and waist circumference was associated with inflammation [12]. Body fat, skinfold thickness, and other measures of abdominal adiposity were also positively correlated with inflammation [13, 14]. Furthermore, metabolic disorders might interfere with inflammatory status. Components of metabolic syndrome were significantly increased with both CRP and neutrophil-to-lymphocyte ratio (NLR) levels [15, 16]. Dyslipidemia, which is characterized by high levels of total cholesterol (TC), serum TG, low-density lipoprotein-cholesterol (LDL-C) levels, or low HDL-C levels, has also been positively correlated with elevated plasma CRP levels, soluble intracellular adhesion molecule (sICAM)-1, and soluble endothelial selectin [17].

Several studies have investigated the effect of dietary patterns, anthropometric measurements, or metabolic parameters on inflammatory markers [18, 19]. However, the study investigated all these factors in metabolic syndrome population using CRP and NLR as the indicators of inflammation was still rare. Both CRP and NLR serve as inflammatory indicators that can be easily measured and serve as independent predictors for both the development of metabolic syndrome and CVD [20]. Thus, the objective of the study was to determine the association of dietary patterns, anthropometric measurements, and metabolic parameters with inflammatory markers using CRP and NLR among middle-aged and older adults with metabolic syndrome in Taiwan.

## Methods

### Subjects and study design

The cross-sectional study was performed to examine data collected from a Mei Jau (MJ) Group, a private health management screening institution in Taiwan, from 2004 to 2013. The MJ Group’s four health screening centers in Taiwan in Taipei, Taoyuan, Taichung, and Kaohsiung (listed from north to south) were used to collect pertinent information for the study. Data collected included demographic data, lifestyle, diet, anthropometric data, biochemical parameters, and other health related data from the individuals who came to their health screening centers for a regular health check-up [9]. A total of 60,769 individuals with age ≥ 35 y met criteria of metabolic syndrome from the MJ database between 2004 and 2013. After excluding the individuals (*n* = 23,377) who had renal dysfunction, liver problems, or all types of cancer, and those who (*n* = 11,376) with missing data on food frequency questionnaire (FFQ), anthropometric measurements, or biochemical parameters, a total of 26,016 subjects were finally recruited for analysis in this study.

### Definition of metabolic syndrome and inflammation

The components of metabolic syndrome were proposed by the IDF in 2005 and defined as individuals who had central obesity (waist circumference ≥ 90 cm for men or ≥ 80 cm for women in Taiwan) with two of four factors: (1) systolic BP ≥ 130 mmHg, diastolic BP ≥ 85 mmHg or history of the therapy of hypertension, (2) HDL-C < 1.03 mmol/L for men, < 1.29 mmol/L for women or specific therapy for lipid abnormality, (3) serum TG ≥ 1.70 mmol/L or specific therapy for lipid abnormality, and (4) FBG ≥ 5.60 mmol/L or previously diagnosed type 2 diabetes mellitus [1]. The definition of metabolic syndrome with central obesity was selected due to the growing prevalence of central obesity in Taiwan in recent years [9]. The definition of inflammation in this study was CRP ≥ 28.6 nmol/L [21] or NLR ≥ 3.0 [22]. Ethical approval for this study was granted by Taipei Medical University-Joint Institutional Review Board (N201706051). Written informed consents were obtained from all subjects prior to the health check-up when they visited each MJ Group health screening center. The data were de-identified and used for academic study only.

### Assessment of dietary patterns

Dietary intake was analyzed using a validated FFQ collected by the MJ Group. All subjects were requested to complete the FFQ before they had health check-up. The FFQ included 22 food groups or food items referred to the characteristics of Taiwanese dietary patterns. The FFQ collected intake frequency data with the information of portion size and the corresponding pictures of measuring tools in each question. For instance, the description for milk consumption was “How much milk do you drink? (1 cup is equivalent to 240 mL of fresh milk, 240 mL of yogurt, or 4 tablespoons of powdered milk)”, and the options for intake frequency were “none or < 1 cup a week, 1-3 cups a week, 4-6 cups a week, 1 cup a day, or ≥ 2 cups a day”. Each question had 5 options for intake frequency from the lowest to the highest. Food intake scores of 1 to 5 were assigned from the lowest to the highest frequency. Principal component analysis was performed to derive dietary patterns. We used the eigenvalues ≥2 in the orthogonal rotation to derive dietary patterns, and factor loading ≥0.30 to classify dietary patterns. The eigenvalues ≥2 have been used in the previous studies, and represented a strong correlation between each component and smaller variance compared with the eigenvalue < 2 [23, 24]. The factor scores of dietary patterns were calculated using the sum of food intake scores divided by factor loadings [9], and were classified into tertiles for each dietary pattern.

### Anthropometric measurements

The assessments of anthropometric data were carried out by the medical staff in the MJ health screening centers. Weight (kg), height (cm), and body fat (%) were measured by using a bioelectrical impedance analysis instrument (InBody Co., Ltd., Seoul, South Korea). BMI was computed as weight (kg) divided by the square of height (m^2^), and classified as underweight, normal weight, overweight, or obesity (BMI < 18.5 kg/m^2^, 18.5 kg/m^2^ ≤ BMI < 24 kg/m^2^, 24 kg/m^2^ ≤ BMI < 27 kg/m^2^, and BMI ≥ 27 kg/m^2^, respectively) [25]. Body fat was defined as low (< 25% for men and < 30% for women) and high (≥ 25% for men and ≥ 30% for women) [26–28]. Waist circumference was assessed at the mid-point between the lowest rib and the iliac crest on standing position, while hip circumference was assessed at the point generating the maximum circumference in the buttocks using a measuring tape. We used the mean of waist or hip circumference as the cut-off point to dichotomize these two variables into low (waist circumference < 95.8 cm for men and < 85.2 cm for women, hip circumference < 100.5 cm for men and < 101.1 cm for women) and high (waist circumference ≥ 95.8 cm for men and ≥ 85.2 cm for women, hip circumference ≥ 100.5 cm for men and ≥ 101.1 cm for women). Waist-to-hip ratio was calculated and defined as low (< 0.90 for men and < 0.85 for women) and high (≥ 0.90 for men and ≥ 0.85 for women) [29].

### Blood pressure and biochemical measurements

Blood samples were collected after overnight fasting for 12–14 h. BP was measured in a sitting position using a sphygmomanometer. Blood HDL-C, TC, TG, FBG, and CRP levels were analyzed using the commercial reagents or kits, and LDL-C levels were calculated using Friedewald’s formula: LDL-C (mg/dL) = TC-(HDL-C + TG/5). The number of neutrophils and lymphocytes was determined, and NLR was calculated using the absolute neutrophil count divided by the absolute lymphocyte count. The IDF definition was used to define metabolic syndrome to categorize systolic BP (low < 130 mmHg and high ≥130 mmHg), diastolic BP (low < 85 mmHg and high ≥85 mmHg), HDL-C (low < 1.03 mmol/L and high ≥1.03 mmol/L for men, low < 1.29 mmol/L and high ≥1.29 mmol/L for women), TG (low < 1.70 mmol/L and high ≥1.70 mmol/L), and FBG (low < 5.60 mmol/L and high ≥5.60 mmol/L) [1]. Whereas TC and LDL-C levels were defined as low (< 6.2 mmol/L for TC and < 4.1 mmol/L for LDL-C) and high (≥ 6.2 mmol/L for TC and ≥ 4.1 mmol/L for LDL-C) [30].

### Covariates

Demographic and lifestyle characteristics such as sex, age, marital status, education, occupation, current smoking or drinking status, and physical activity were collected using an administered questionnaire from the MJ Group. Education level was dichotomized as low (high school and below) and high (above high school). Physical activity was categorized as low (< 1 h a week), moderate (1–2 h a week), and high (> 2 h a week).

### Statistical analysis

Chi-square test and general linear model test were used to determine the differences of categorical and continuous variables, respectively, in the characteristics of the subjects with low or high CRP and NLR levels. Odds ratios (OR) with 95% confidence interval were derived using multivariate logistic regression analysis to compare the association of dietary patterns, anthropometric status, and metabolic parameters with CRP and NLR levels in men and women. Model 1 was unadjusted and model 2 was adjusted for age, marital status, education, occupation, smoking, drinking status, and physical activity. A significance level of *P* ≤ 0.05 was used for all analyses, and SPSS 24 (IBM Corp., Armonk, NY, USA) software was used to analyze the data.

## Results

Among 26,016 subjects with metabolic syndrome, 4639 (27.5%), 6650 (39.4%), 4089 (24.2%), 5687 (33.7%), 8933 (52.9%), and 11,903 (70.5%) subjects had high waist circumference (≥ 95.8 cm), high systolic BP, high diastolic BP, low HDL-C, high TG, and high FBG levels, respectively, among men, and 5131 (56.2%), 4139 (46.4%), 1658 (18.2%), 2785 (30.5%), 4155 (45%), and 6333 (69.4%) subjects had high waist circumference (≥ 85.2 cm), high systolic BP, high diastolic BP, low HDL-C, high TG, and high FBG levels, respectively, among women (data not shown).

### Dietary patterns

Two dietary patterns were derived from principal component analysis (Table 1) and defined as the western and prudent dietary patterns. The western and prudent dietary patterns had 12 and 9 food groups or food items, respectively. Legumes or soy products and seafood had factor loading ≥0.30 in both factors, and had higher factor loadings in the prudent dietary pattern. Therefore, we classified both food groups in the prudent dietary pattern. The western dietary pattern was reflected as high intake of deep-fried food, processed food (e.g. sausage, ham, and canned food), sugary drinks, meat (e.g. beef, lamb, pork, veal, chicken and duck), sauce (e.g. pepper salt, ketchup, vinegar, hot sauce and soy sauce), eggs (e.g. chicken, duck and quail eggs), organ meats (e.g. kidneys, intestines, liver and heart), rice or flour cooked in oil (e.g. fried noodle and rice noodle), instant noodle, jam or honey, rice or flour products (e.g. rice, plain bread, noodle and cruller), and refined desert; while the prudent dietary pattern was reflected by high intake of dark-colored vegetables (e.g. spinach, squash, carrot and tomato), light-colored vegetables (e.g. cabbage, pechay, cucumber and radish), vegetables with oil or dressing, fruit, legumes or soy products (e.g. soybean milk, tofu and dried bean curd), milk (e.g. powdered and fresh milk), dairy products (e.g. cheese and yoghurt), root crops (e.g. corn, potato and taro), and seafood (e.g. fish, shells, oysters and shrimps). The western and prudent dietary patterns had a total variance of 26.3% (16.9 and 9.4%, respectively) and eigenvalues > 2. The two dietary patterns were similar to the unhealthy and healthy dietary patterns, respectively.

**Table 1:** Factor loadings and dietary patterns derived from principal component analysis of food frequency questionnaire data

Food Groups	Factor I Western dietary pattern	Factor II Prudent dietary pattern
Milk	−0.045	0.380
Dairy products	0.166	0.377
Eggs	0.502	0.112
Meat	0.531	0.131
Organ meats	0.500	0.110
Legumes/soy products	0.306	0.390
Seafood	0.316	0.363
Light-colored vegetables	−0.039	0.799
Dark-colored vegetables	−0.057	0.831
Fruit	−0.014	0.521
Vegetables with oil/dressing	0.261	0.524
Rice/flour products	0.381	0.143
Whole grains	0.071	0.228
Root crops	0.201	0.369
Refined dessert	0.319	0.131
Jam/honey	0.382	0.108
Sugary drinks	0.542	−0.098
Rice/flour cooked in oil	0.436	0.066
Deep-fried food	0.619	0.044
Instant noodle	0.392	−0.083
Processed food	0.586	0.036
Sauce	0.526	0.071

### Characteristics of subjects

The characteristics of subjects with low or high CRP and NLR in both genders are summarized in Table 2. There were 10,096 (38.8%) and 7857 (30.2%) subjects with high CRP and NLR, respectively. Among those who had high CRP or NLR, the proportion of men was 59.6 and 65.6%, respectively. The majority of the subjects with high CRP or NLR were between the ages of 46 and 60 years (55.6 and 55.6%), were married (84.3 and 85.5%), had education level of high school and below (54.1 and 52.9%), had professional jobs (43.6 and 45.2%), did not smoke (95.0 and 95.5%), did not drink alcohol (94.0 and 94.2%), and had low physical activity (51.2 and 49.6%). The results also showed that subjects with high CRP or NLR were more likely to consume the highest tertile in the western dietary pattern (35.2 and 35.6%) and the lowest tertile in the prudent dietary pattern (36.0 and 34.4%). Subjects with high CRP or NLR had significantly higher BMI (27.6 ± 2.8 and 27.4 ± 2.6 kg/m^2^), body fat (28.0 ± 4.7% and 27.6 ± 4.6%), waist circumference (94.4 ± 8.6 and 94.6 ± 8.1 cm), hip circumference (101.5 ± 6.7 and 101.3 ± 6.3 cm), and waist-to-hip ratio (0.93 ± 0.07 and 0.94 ± 8.13) than those who with low CRP or NLR. Additionally, subjects with high CRP or NLR had significantly higher systolic BP (124 ± 33 and 125 ± 32 mmHg), diastolic BP (75 ± 19 and 75 ± 19 mmHg), LDL-C (3.75 ± 0.87 and 3.73 ± 0.89 mmol/L), TC (5.29 ± 0.89 and 5.30 ± 0.92 mmol/L), TG (2.17 ± 1.34 and 2.09 ± 1.30 mmol/L), and FBG (6.76 ± 2.43 and 6.67 ± 2.29 mmol/L) but lower HDL-C (1.12 ± 0.31 and 1.15 ± 0.07 mmol/L) than those who with low CRP or NLR.

**Table 2:** Characteristics of subjects across values of C-reactive protein and neutrophil-to-lymphocyte ratio (*n* = 26,016) ^a^

Variables	C-reactive protein		*P* ^b^	Neutrophil-to-lymphocyte ratio		*P* ^b^
	< 28.6 nmol/L	≥ 28.6 nmol/L		< 3.0	≥ 3.0	
Sex, %			0.000			0.049
Men	68.2	59.6		64.6	65.6	
Women	31.8	40.4		35.4	34.4	
Age, %			0.205			0.973
35–45 y	15.8	15.0		15.4	15.5	
46–60 y	55.4	55.6		55.4	55.6	
> 60 y	28.9	29.4		29.2	28.9	
Marital status, %			0.043			0.009
Not married	3.0	3.5		3.2	3.0	
Married	84.6	84.3		84.0	85.5	
Divorced	12.4	12.2		12.7	11.5	
Education, %			0.000			0.247
Low	51.7	54.1		52.5	52.9	
High	48.3	45.9		47.5	47.1	
Occupation, %			0.000			0.004
Professional	48.3	43.6		47.0	45.2	
Not professional	31.2	35.7		32.9	33.0	
Unemployed/retired	20.5	20.7		20.1	21.8	
Current smoking, %			0.000			0.000
No	96.9	95.0		96.4	95.5	
Yes	3.1	5.0		3.6	4.5	
Current drinking status, %			0.000			0.000
No	96.4	94.0		95.9	94.2	
Yes	3.6	6.0		4.1	5.8	
Physical activity, %			0.000			0.000
Low	48.8	51.2		41.3	49.6	
Moderate	45.5	42.3		52.8	43.9	
High	5.8	6.5		5.9	6.4	
Western dietary pattern, %			0.000			0.000
T1, *n*	34.4	31.7		34.7	30.2	
T2, *n*	33.5	33.1		32.9	34.2	
T3, *n*	32.1	35.2		32.4	35.6	
Prudent dietary patterns, %			0.000			0.000
T1, *n*	31.7	36.0		32.9	34.4	
T2, *n*	31.4	36.2		32.8	34.5	
T3, *n*	36.9	27.8		34.3	31.1	
Body mass index, kg/m^2^	26.9 ± 2.3	27.6 ± 2.8	0.000	27.0 ± 2.4	27.4 ± 2.6	0.000
Body fat, %	26.7 ± 4.3	28.0 ± 4.7	0.000	27.0 ± 4.4	27.6 ± 4.6	0.000
Waist circumference, cm	92.0 ± 6.5	94.4 ± 8.6	0.000	92.1 ± 6.9	94.6 ± 8.1	0.000
Hip circumference, cm	100.0 ± 5.9	101.5 ± 6.7	0.000	100.2 ± 6.1	101.3 ± 6.3	0.000
Waist-to-hip ratio	0.92 ± 0.07	0.93 ± 0.07	0.000	0.92 ± 0.06	0.94 ± 8.13	0.000
Systolic blood pressure, mmHg	120 ± 30	124 ± 33	0.000	120 ± 31	125 ± 32	0.000
Diastolic blood pressure, mmHg	74 ± 20	75 ± 19	0.000	74 ± 20	75 ± 19	0.000
High-density lipoprotein-cholesterol, mmol/L	1.19 ± 0.34	1.12 ± 0.31	0.000	1.17 ± 0.34	1.15 ± 0.07	0.026
Low-density lipoprotein-cholesterol, mmol/L	3.61 ± 0.83	3.75 ± 0.87	0.000	3.62 ± 0.83	3.73 ± 0.89	0.000
Total cholesterol, mmol/L	5.19 ± 0.87	5.29 ± 0.89	0.000	5.20 ± 0.86	5.30 ± 0.92	0.000
Triglycerides, mmol/L	1.96 ± .37	2.17 ± 1.34	0.000	2.01 ± 1.39	2.09 ± 1.30	0.000
Fasting blood glucose, mmol/L	6.11 ± 1.54	6.76 ± 2.43	0.000	6.20 ± 1.73	6.67 ± 2.29	0.000

^a^Data are presented as % for categorical variables and mean ± SD for continuous variables

^b^*P*-values were derived from chi-square test for categorical variables and general linear regression for continuous variables

### Dietary patterns, nutritional status and levels of CRP and NLR in men and women

The results showed that regardless of gender, subjects who had higher tertiles (T2 and T3) of the western dietary pattern, overweight or obesity, high body fat, high waist or hip circumference, high waist-to-hip ratio, high systolic or diastolic BP, low HDL-C, high LDL-C, high TC, high TG, or high FBG had significantly increased odds ratios of high CRP (≥ 28.6 nmol/L) in both models (Table 3). However, higher tertiles (T2 and T3) of the prudent dietary pattern were significantly associated with decreased odds ratios of high CRP in men and women in both models.

**Table 3:** Odds ratios for high C-reactive protein by different dietary patterns, anthropometric measurements, and metabolic parameters

Variables	Odds ratio (95% Confidence interval)			
	Men (*n* = 6021)		Women (*n* = 4075)	
	Model 1^1^	Model 2^2^	Model 1^1^	Model 2^2^
Western dietary pattern				
T1	1	1	1	1
T2	0.892 (0.843–0.944) ^***^	0.903 (0.853–0.956) ^***^	0.843 (0.778–0.913) ^***^	0.855 (0.788–0.927) ^***^
T3	1.116 (1.053–1.184) ^***^	1.108 (1.044–1.176) ^**^	1.162 (1.070–1.263) ^***^	1.181 (1.086–1.284) ^***^
Prudent dietary pattern				
T1	1	1	1	1
T2	0.810 (0.765–0.857) ^***^	0.815 (0.770–0.863) ^***^	0.886 (0.824–0.954) ^**^	0.891 (0.827–0.959) ^**^
T3	0.693 (0.654–0.735) ^***^	0.696 (0.656–0.738) ^***^	0.814 (0.756–0.877) ^***^	0.806 (0.748–0.869) ^***^
Body mass index				
Normal	1	1	1	1
Overweight	1.230 (1.147–1.320) ^***^	1.225 (1.141–1.315) ^***^	1.149 (1.036–1.275) ^**^	1.182 (1.065–1.313) ^**^
Obese	1.315 (1.190–1.454) ^***^	1.328 (1.199–1.470) ^***^	1.961 (1.702–2.260) ^***^	1.971 (1.709–2.273) ^***^
Body fat				
Low	1	1	1	1
High	1.734 (1.613–1.864) ^***^	1.749 (1.624–1.884) ^***^	1.454 (1.023–2.068) ^*^	1.413 (0.981–2.028) ^*^
Waist circumference				
Low	1	1	1	1
High	1.963 (1.826–2.110) ^***^	1.916 (1.782–2.061) ^***^	1.986 (1.820–2.168) ^***^	2.003 (1.834–2.188) ^***^
Hip circumference				
Low	1	1	1	1
High	1.422 (1.264–1.599) ^***^	1.406 (1.247–1.587) ^***^	1.637 (1.499–1.787) ^***^	1.651 (1.510–1.805) ^***^
Waist-to-hip ratio				
Low	1	1	1	1
High	1.487 (1.384–1.598) ^***^	1.488 (1.383–1.601) ^***^	1.298 (1.184–1.423) ^***^	1.307 (1.190–1.436) ^***^
Systolic blood pressure				
Low	1	1	1	1
High	1.258 (1.176–1.345) ^***^	1.280 (1.195–1.371) ^***^	1.294 (1.188–1.409) ^***^	1.306 (1.196–1.425) ^***^
Diastolic blood pressure				
Low	1	1	1	1
High	1.303 (1.208–1.405) ^***^	1.303 (1.208–1.406) ^***^	1.306 (1.170–1.457) ^***^	1.313 (1.175–1.467) ^***^
High-density lipoprotein-cholesterol				
High	1	1	1	1
Low	1.625 (1.517–1.740) ^***^	1.635 (1.525–1.753) ^***^	1.379 (1.257–1.512) ^***^	1.419 (1.292–1.558) ^***^
Low-density lipoprotein-cholesterol				
Low	1	1	1	1
High	1.278 (1.191–1.373) ^***^	1.276 (1.188–1.371) ^***^	1.239 (1.132–1.356) ^***^	1.250 (1.141–1.369) ^***^
Total cholesterol				
Low	1	1	1	1
High	1.157 (1.083–1.237) ^***^	1.148 (1.074–1.227) ^***^	1.228 (1.123–1.342) ^***^	1.225 (1.118–1.342) ^***^
Triglycerides				
Low	1	1	1	1
High	1.493 (1.397–1.595) ^***^	1.475 (1.379–1.578) ^***^	1.717 (1.576–1.871) ^***^	1.694 (1.553–1.847) ^***^
Fasting blood glucose				
Low	1	1	1	1
High	1.366 (1.270–1.470) ^***^	1.398 (1.297–1.506) ^***^	1.257 (1.146–1.380) ^***^	1.245 (1.133–1.368) ^***^

^1^Unadjusted. ^2^Adjusted for age, marital status, education, occupation, smoking, drinking status, and physical activity. ^*^P < 0.05, ^**^P < 0.01, ^***^P < 0.001. The variables were defined as the following: CRP: low < 28.6 nmol/L and high ≥28.6 nmol/L, normal BMI: 18.5 kg/m^2^ ≤ BMI < 24 kg/m^2^; overweight BMI: 24 kg/m^2^ ≤ BMI < 27 kg/m^2^; obesity BMI: ≥ 27 kg/m^2^, body fat: low < 25% and high ≥25% for men; low < 30% and high ≥30% for women, waist circumference: low < 95.8 cm and high ≥95.8 cm for men; low < 85.2 cm and high ≥85.2 cm for women, hip circumference: low < 100.5 cm and high ≥100.5 cm for men; low < 101.1 cm and high ≥101.1 cm for women, waist-to-hip ratio: low < 0.90 and high ≥0.90 for men; low < 0.85 and high ≥0.85 for women, systolic BP: low < 130 mmHg and high ≥130 mmHg, diastolic BP: low < 85 mmHg and high ≥85 mmHg, HDL-C: low < 1.03 mmol/L and high ≥1.03 mmol/L for men; low < 1.29 mmol/L and high ≥1.29 mmol/L for women, LDL-C: low < 4.1 mmol/L and high ≥4.1 mmol/L, TC: low < 6.2 mmol/L and high ≥6.2 mmol/L, TG: low < 1.70 mmol/L and high ≥1.70 mmol/L, FBG: low < 5.60 mmol/L and high ≥5.60 mmol/L

Furthermore, subjects who had the highest tertile (T3) of the western dietary pattern, obesity, high body fat, high waist or hip circumference, high waist-to-hip ratio, low HDL-C, high LDL-C, high TG, or high FBG had significantly increased odds ratios of high NLR (≥ 3.0) in men and women in both models (Table 4). However, the highest tertile (T3) of the prudent dietary pattern was significantly correlated with reduced odds ratios of high NLR (≥ 3.0) in men and women in both models. Only men with high systolic or diastolic BP or high TC had increased odds ratios of high NLR (≥ 3.0) in both models.

**Table 4:** Odds ratios for high neutrophil-to-lymphocyte ratio by different dietary patterns, anthropometric measurements, and metabolic parameters

Variables	Men (*n* = 5155)		Women (*n* = 2702)	
	Model 1^1^	Model 2^2^	Model 1^1^	Model 2^2^
Western dietary pattern				
T1	1	1	1	1
T2	0.955 (0.900–1.013)	0.963 (0.908–1.022)	0.941 (0.867–1.021)	0.945 (0.871–1.026)
T3	1.189 (1.118–1.264) ^***^	1.199 (1.126–1.275) ^***^	1.100 (1.012–1.195) ^*^	1.108 (1.019–1.204) ^*^
Prudent dietary pattern				
T1	1	1	1	1
T2	0.916 (0.864–0.972) ^**^	0.921 (0.868–0.977) ^**^	1.134 (1.046–1.228)	1.111 (1.023–1.205)
T3	0.881 (0.830–0.935) ^***^	0.883 (0.832–0.938) ^***^	0.961 (0.873–1.056) ^*^	0.963 (0.874–1.059) ^*^
Body mass index				
Normal	1	1	1	1
Overweight	1.154 (1.073–1.241) ^***^	1.141 (1.061–1.227) ^***^	1.081 (0.978–1.195)	1.096 (0.998–1.212)
Obese	1.162 (1.049–1.288) ^**^	1.188 (1.070–1.319) ^**^	1.575 (1.375–1.804) ^***^	1.576 (1.375–1.806) ^***^
Body fat				
Low	1	1	1	1
High	1.276 (1.186–1.374) ^***^	1.286 (1.192–1.386) ^***^	1.301 (1.143–1.480) ^***^	1.158 (1.015–1.323) ^*^
Waist circumference				
Low	1	1	1	1
High	1.711 (1.588–1.843) ^***^	1.690 (1.568–1.822) ^***^	1.468 (1.335–1.614) ^***^	1.475 (1.341–1.623) ^***^
Hip circumference				
Low	1	1	1	1
High	1.185 (1.052–1.334) ^**^	1.201 (1.063–1.358) ^**^	1.259 (1.145–1.386) ^***^	1.275 (1.157–1.404) ^***^
Waist-to-hip ratio				
Low	1	1	1	1
High	1.626 (1.506–1.754) ^***^	1.630 (1.509–1.761) ^***^	1.336 (1.211–1.474) ^***^	1.331 (1.204–1.472) ^***^
Systolic blood pressure				
Low	1	1	1	1
High	1.122 (1.047–1.203) ^**^	1.124 (1.047–1.206) ^**^	1.037 (0.946–1.138)	1.038 (0.944–1.142)
Diastolic blood pressure				
Low	1	1	1	1
High	1.182 (1.092–1.278) ^***^	1.176 (1.087–1.273) ^***^	0.946 (0.839–1.067)	0.928 (0.821–1.049)
High-density lipoprotein-cholesterol				
High	1	1	1	1
Low	1.197 (1.114–1.286) ^***^	1.203 (1.119–1.294) ^***^	1.138 (1.031–1.257) ^*^	1.162 (1.051–1.286) ^**^
Low-density lipoprotein-cholesterol				
Low	1	1	1	1
High	1.197 (1.112–1.289) ^***^	1.200 (1.114–1.293) ^***^	1.105 (1.002–1.219) ^*^	1.113 (1.008–1.229) ^*^
Total cholesterol				
Low	1	1	1	1
High	1.138 (1.063–1.219) ^***^	1.138 (1.062–1.220) ^***^	1.070 (0.971–1.179)	1.069 (0.968–1.180)
Triglycerides				
Low	1	1	1	1
High	1.262 (1.179–1.352) ^***^	1.251 (1.167–1.342) ^***^	1.157 (1.055–1.269) ^**^	1.140 (1.039–1.252) ^**^
Fasting blood glucose				
Low	1	1	1	1
High	1.294 (1.199–1.396) ^***^	1.300 (1.203–1.405) ^***^	1.199 (1.083–1.328) ^***^	1.180 (1.064–1.308) ^**^

^1^Unadjusted. ^2^ Adjusted for age, marital status, education, occupation, smoking, drinking status and physical activity. ^*^P < 0.05, ^**^P < 0.01, ^***^P < 0.001. The variables were defined as the following: NLR: low < 3.0 and normal ≥3.0; normal BMI: 18.5 kg/m^2^ ≤ BMI < 24 kg/m^2^; overweight BMI: 24 kg/m^2^ ≤ BMI < 27 kg/m^2^; obesity BMI: ≥ 27 kg/m^2^, body fat: low < 25% and high ≥25% for men; low < 30% and high ≥30% for women, waist circumference: low < 95.8 cm and high ≥95.8 cm for men; low < 85.2 cm and high ≥85.2 cm for women, hip circumference: low < 100.5 cm and high ≥100.5 cm for men; low < 101.1 cm and high ≥101.1 cm for women, waist-to-hip ratio: low < 0.90 and high ≥0.90 for men; low < 0.85 and high ≥0.85 for women, systolic BP: low < 130 mmHg and high ≥130 mmHg, diastolic BP: low < 85 mmHg and high ≥85 mmHg, HDL-C: low < 1.03 mmol/L and high ≥1.03 mmol/L for men; low < 1.29 mmol/L and high ≥1.29 mmol/L for women, LDL-C: low < 4.1 mmol/L and high ≥4.1 mmol/L, TC: low < 6.2 mmol/L and high ≥6.2 mmol/L, TG: low < 1.70 mmol/L and high ≥1.70 mmol/L, FBG: low < 5.60 mmol/L and high ≥5.60 mmol/L

## Discussion

Our main findings are that dietary patterns, anthropometric measurements, and metabolic parameters were directly associated with CRP and NLR among Taiwanese men and women aged 35 and above with metabolic syndrome. Subjects who consumed more western dietary pattern were positively correlated with inflammation, while subjects who consumed more prudent dietary pattern were inversely associated with inflammation in both genders. Moreover, anthropometric measurements and metabolic parameters were strongly associated with CRP and NLR in both genders.

The present study revealed that subjects with the high levels of inflammatory markers were more frequent in men and less active physically compared with those with low levels of inflammatory markers. The findings were consistent with the results in the previous studies [16, 31]. The western dietary pattern in this study was similar to the unhealthy dietary pattern that included a high relative amount of red meat, processed food, high-fat food, sweets, salts, and food additives [8]. This dietary pattern was significantly correlated with increased CRP and NLR levels in the subjects with metabolic syndrome. The previous study found that an unhealthy dietary pattern was positively associated with the levels of inflammatory markers such as CRP, interleukin-6 and sICAM-1 [32]. Additionally, there was a positive association between western dietary pattern and CRP concentration [9, 33]. An animal study also revealed that neutrophil counts were increased in mice fed a high-fat diet [34]. In contrast with the unhealthy dietary pattern, the prudent dietary pattern in this study was defined by foods high in complex carbohydrate, unsaturated fat, fiber, antioxidants, vitamins, and minerals. Moreover, a healthy dietary pattern had beneficial effects on inflammation [35]. The prudent breakfast high in dietary fiber and β-glucan for 12 weeks improved plasma CRP in overweight and mildly hypercholesterolemic adults aged of 25–67 years compared with the usual breakfast [36]. Furthermore, the Mediterranean diet characterized as high in fiber, antioxidants, and unsaturated and polyunsaturated fatty acids [37] was correlated with decreases in platelet count, white blood cells (WBC) count and CRP [38, 39]. Our findings showed that there were no differences between men and women in the effects of the western or prudent dietary pattern on CRP and NLR levels. Similar to our results, following the Mediterranean diet for 4 weeks, had similar effects on high-sensitivity CRP levels in mildly hypercholesterolemic men and women aged of 24–53 years with CVD risk factors [40], indicating there were no differences between men and women in the effects of the Mediterranean diet on systemic inflammation.

Our results reported that the anthropometric measures such as BMI, body fat, waist or hip circumference, and waist-to-hip ratio were associated with inflammation in both men and women with metabolic syndrome. Some evidences supported our findings. BMI was independently correlated with certain markers related to inflammatory responses, including CRP, amylin, C-peptide, insulin, leptin, WBC, and NLR [41, 42]. The indicators of central obesity such as waist or hip circumference and waist-to-hip ratio showed a strong positive correlation with CRP not only in healthy population, but also in the population with metabolic syndrome [43, 44]. Moreover, waist circumference was increased with elevated WBC and NLR, suggesting that waist circumference might be used as a parameter of evaluating WBC or NLR [45]. Body fat and BMI were also significantly associated with CRP in obese males and females with metabolic syndrome and heart failure [46]. Additionally, obese subjects had higher NLR compared with healthy subjects, and elevated NLR considered as an inflammatory marker was an independent predictor of type 2 diabetes in obese subjects [47]. Furthermore, this study found that more than 30% of subjects with metabolic syndrome had higher levels of CRP (38.8%) or NLR (30.2%). The previous studies also revealed that the components of metabolic syndrome influenced the levels of CRP and NLR [48, 49]. Subjects with metabolic syndrome had significantly increased neutrophil counts, but reduced lymphocyte counts [50]. Hypercholesterolemia was one of the major risk factors of elevated pro-inflammatory cytokines. Hyperlipidemia had effects on homeostasis of immune cells and was associated with increased neutrophil counts [51]. The levels of CRP were positively correlated with fasting and 2-h post-load glucose concentrations in individuals with impaired glucose tolerance [52], and individuals with prediabetes also had higher CRP levels [53]. The potential interaction among obesity, metabolic disorder, and activated inflammation in subjects with metabolic syndrome has been reported. The association between chronic inflammation and metabolic syndrome was linked to central adiposity, which was accompanied by a decrease of adiponectin formed in adipose tissue and increased secretion of inflammatory markers such as CRP [54].

Our results found that both men and women with high consumption of the western dietary pattern, low consumption of the prudent dietary pattern, high values of anthropometric parameters or metabolic disorder increased the likelihood of being high CRP or NLR. However, there were gender differences in the effects of BP or TC on NLR values. Higher systolic or diastolic BP or TC was significantly associated with increased odds ratios of high NLR in men, but not in women. There is still a conflicting evidence regarding whether NLR is a good indicator of inflammation in a metabolic syndrome population. Neutrophil-to-lymphocyte ratio was not a better indicator of inflammation compared with CRP in obese subjects with metabolic syndrome [55], although both CRP and NLR were simple and effective predictors of inflammation in subjects with metabolic syndrome. CRP as an acute phase protein is a sensitive biomarker for systemic inflammation and correlated significantly with metabolic abnormality [56]. Contrarily, high neutrophil counts play an important role in atherogenesis and atherothrombosis, and low lymphocyte counts have been observed in patients with acute coronary syndrome and its complication [57]. Use of the neutrophil-to-lymphocyte ratio has recently emerged as an alternate potential biomarker for both metabolic syndrome and CVD events [49, 58].

### Strengths and limitations

This study has some strengths. This is the first study to discuss the association of dietary patterns, anthropometric measures and metabolic parameters with inflammatory markers in Taiwanese middle-aged and older adults with metabolic syndrome. In addition, the sample size was large from the population of interest. We defined metabolic syndrome by using the IDF definition, which uses central obesity as an essential component followed by other components of metabolic syndrome. This parameter was appropriate because the prevalence of central obesity or metabolic syndrome increases with age in both genders in Taiwan [5] and was expected to adequately represent the health issues most prevalent among middle-aged and older adults in Taiwan. Some limitations of the study included the cross-sectional study design that limits the applicability of the findings in establishing causality between the variables. Furthermore, there may be some potential confounders in this study that could not be controlled for due to the inherent limitations of nutrition research; such as the intake of energy, protein, and other specific nutrients. The conclusion may not be applied to the whole Taiwanese population with metabolic syndrome, since we only analyzed the subjects aged 35 years and older, and the data from one private health management screening institution with multi-centers in Taiwan.

## Conclusions

Men and women have a similar association of dietary patterns and anthropometric measurements with inflammatory markers. The western dietary pattern is positively correlated with CRP and NLR. In contrast, the prudent dietary pattern is inversely associated with CRP and NLR. Furthermore, better BMI, body fat, waist or hip circumference, and waist-to-hip ratio improve CRP and NLR. Men and women have similar associations between metabolic parameters (systolic and diastolic BP, HDL-C, LDL-C, TC, TG, and FBG) and CRP. However, systolic or diastolic BP and TC show significantly positive correlations with NLR only in men.

## Abbreviations

BMI: Body mass index
BP: Blood pressure
CRP: C-reactive protein
CVD: Cardiovascular disease
FBG: Fasting blood glucose
FFQ: Food frequency questionnaire
HDL-C: High-density lipoprotein-cholesterol
IDF: International Diabetes Federation
LDL-C: Low-density lipoprotein-cholesterol
MJ: Mei Jau
NLR: Neutrophil-to-lymphocyte ratio
OR: Odds ratios
sICAM: Soluble intracellular adhesion molecule
TC: Total cholesterol
TG: Triglycerides
WBC: White blood cells
